# Reimplantation of the Anomalous Origin of the Right Coronary Artery: A Case Report

**DOI:** 10.7759/cureus.99144

**Published:** 2025-12-13

**Authors:** Stephen Maharaj, Amanda McCarthy, Gianni Angelini, Giovanni Teodori, Natasha Rahaman, Risshi D Rampersad

**Affiliations:** 1 Cardiology, Caribbean Heart Care Medcorp, Port-of-Spain, TTO; 2 Cardiac Surgery, Bristol Heart Institute - University of Bristol, Bristol, GBR; 3 Surgery, Caribbean Heart Care Medcorp, Port-of-Spain, TTO

**Keywords:** anomalous origin of right coronary artery, coronary artery anomalies, right coronary artery reimplantation, sinus of valsalva, sudden cardiac death (scd)

## Abstract

Coronary artery anomalies are quite rare, often asymptomatic, but in some cases can be harmful, leading to myocardial infarction and even sudden cardiac death.

We report the case of a 41-year-old male experiencing shortness of breath, angina-like neck tightness, and dizziness when speaking for extended periods. A coronary angiogram revealed that his right coronary artery (RCA) originated from the left coronary sinus of Valsalva (LCSV), coursing anteriorly between the aorta and the main pulmonary artery (malignant interarterial course), but was patent, with no lesions. Following successful open-heart surgery to reimplant the anomalous origin of the RCA, the patient had an uneventful recovery and remains asymptomatic at six-month follow-up.

## Introduction

Coronary artery anomalies are quite rare, with an estimated incidence of 0.1% to 0.9% [1,2]. They are often asymptomatic but can have serious complications, leading to arrhythmias, myocardial infarction, and sudden cardiac death. The anomalous right coronary artery (RCA) commonly originates from the left sinus of Valsalva [2]. An anomalous RCA coursing anteriorly between the aorta and the main pulmonary artery is considered a malignant interarterial course and has a high risk of compression, especially during exercise, which may lead to ischemia and sudden cardiac death [2]. The management of anomalous aortic origin of the right coronary artery (AAORCA) remains a subject of debate, particularly in asymptomatic patients. However, according to the European Society of Cardiology (ESC) Guidelines, a surgical approach for correction should be considered for symptomatic patients or those with high-risk anatomical features [3].

We present the case of a 41-year-old man with no significant medical history, who developed angina-like symptoms during minimal exertion. A coronary angiogram revealed an anomalous RCA arising from the left coronary sinus of Valsalva (LCSV), with an interarterial course. This case highlights the successful surgical reimplantation of the anomalous origin of the RCA. 

## Case presentation

**Figure 1:**
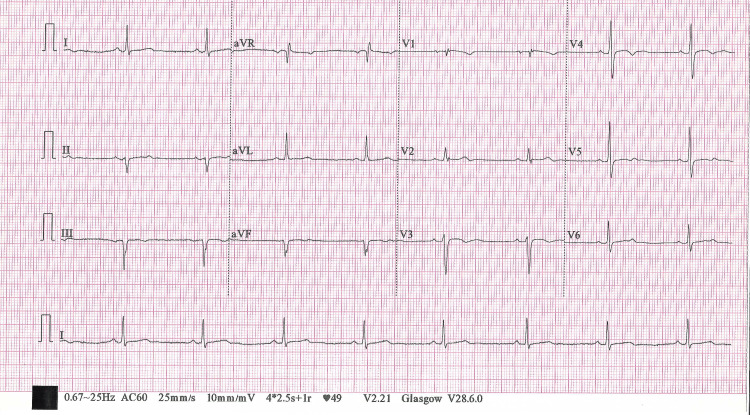
Preoperative electrocardiogram

In December 2023, a 41-year-old male with no significant medical history experienced shortness of breath, angina-like neck tightness, and dizziness when speaking for extended periods. He denies nausea, vomiting, syncope, or classic chest pain. The patient has no significant family history of cardiovascular disease. Electrocardiogram (ECG) showed sinus bradycardia with a left anterior fascicular block (Figure 1). Echocardiogram findings included a normal left ventricular size, overall systolic function, and no significant valvular heart disease, with an ejection fraction of 60%-65%.

**Figure 2:**
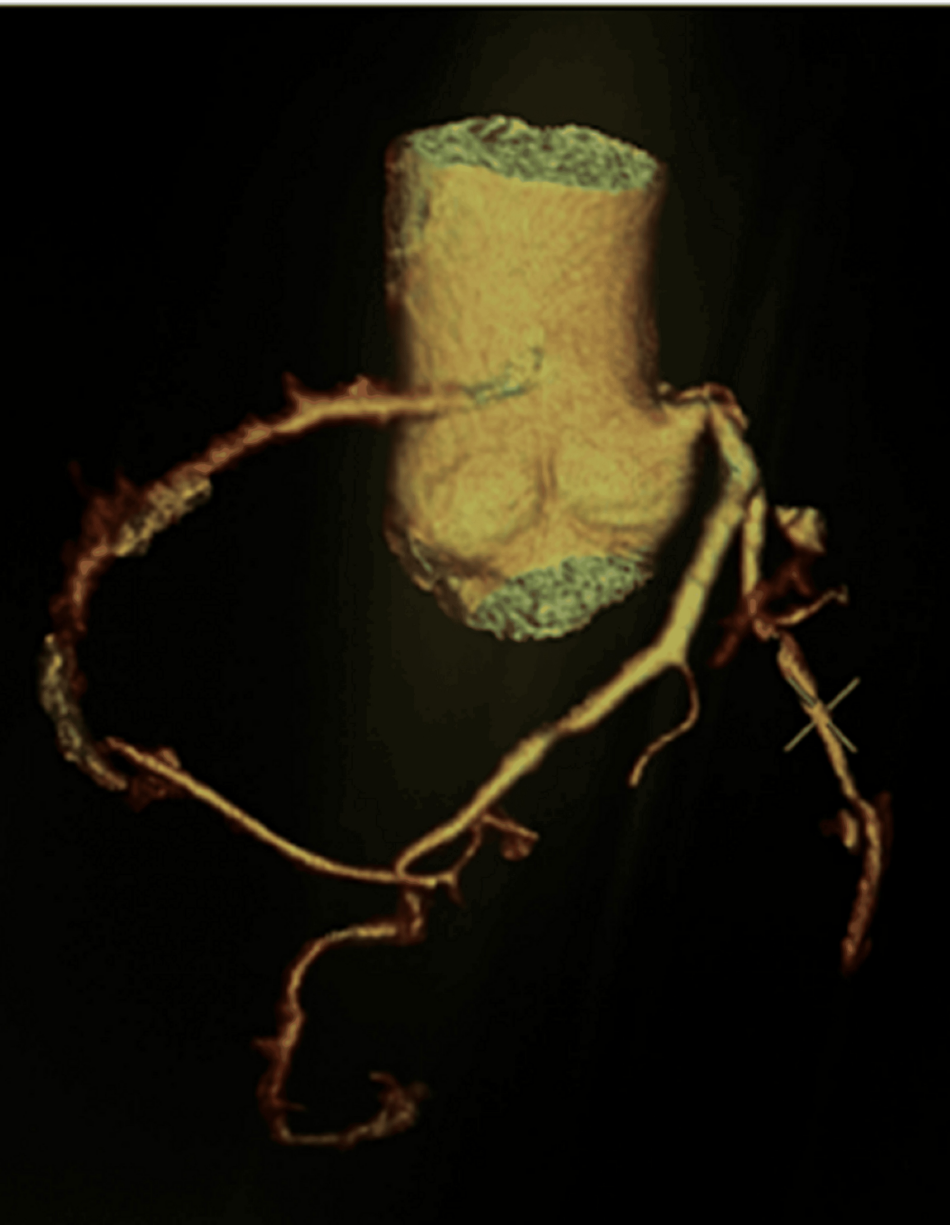
CT coronary angiogram: preoperative three-dimensional reconstruction of an anomalous RCA origin from the left coronary sinus of Valsalva in an isolated aortic view CT, computed tomography; RCA, right coronary artery

**Figure 3:**
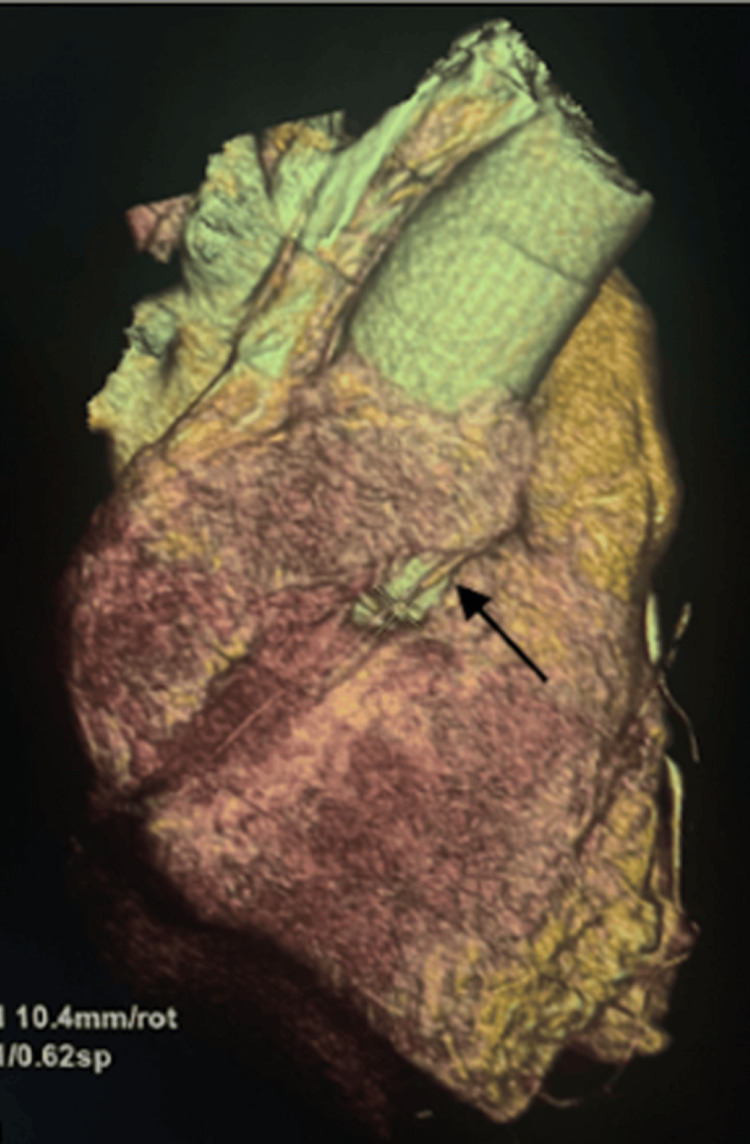
CT coronary angiogram: preoperative three-dimensional reconstruction of an anomalous RCA origin from the left coronary sinus as indicated by the arrow CT, computed tomography; RCA, right coronary artery

**Figure 4:**
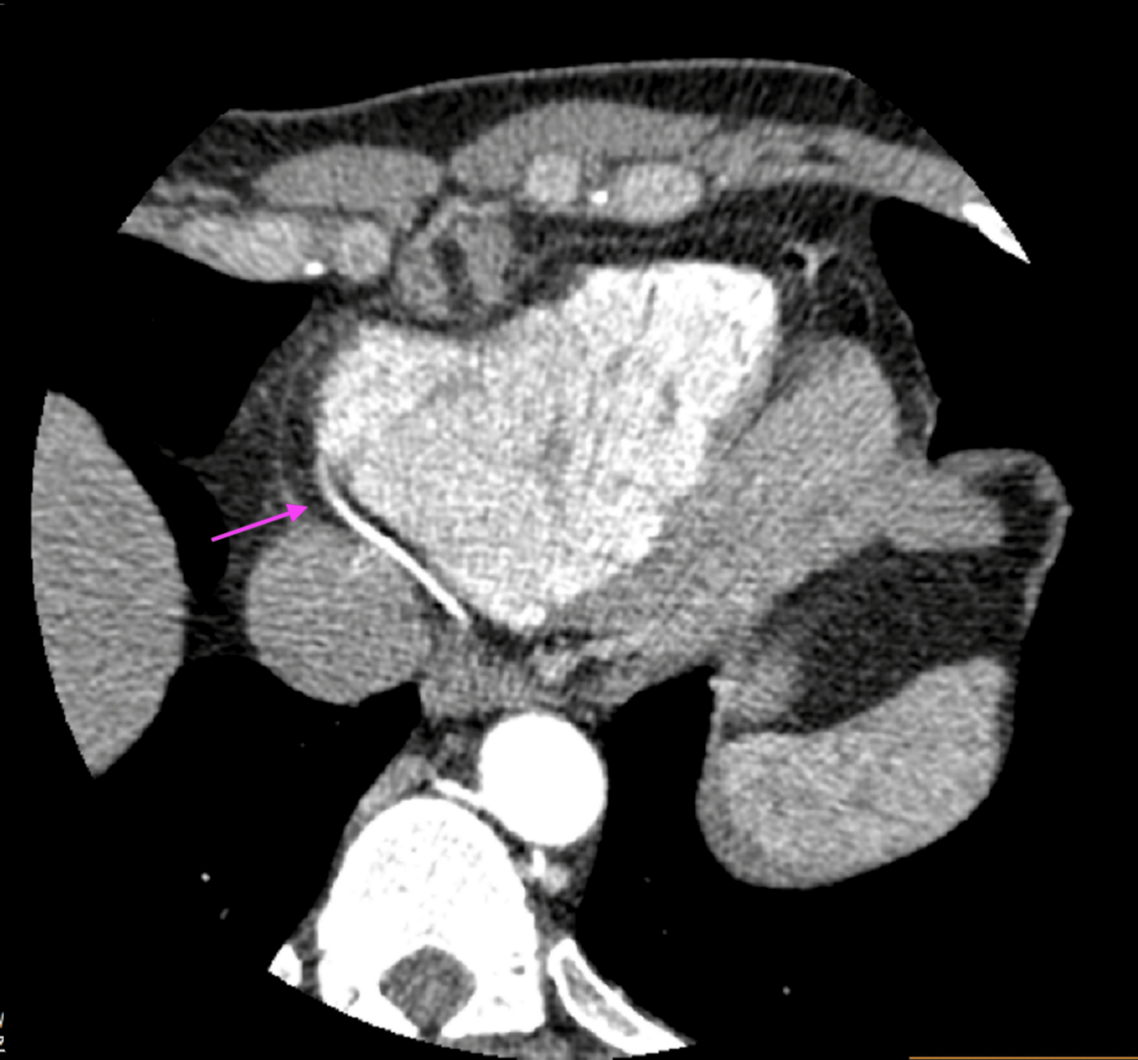
CT angiogram showing the anomalous origin of the RCA CT, computed tomography; RCA, right coronary artery

On further investigation, a coronary angiogram showed that the RCA arose superior to the LCSV, and coursed between the aorta and the main pulmonary artery (malignant interarterial course) (Figures 2-4). The RCA was small in calibre, with a slit-like opening and acute angulation at its origin. The left main coronary artery arose normally from the left sinus of Valsalva.

At this point, he was referred to our hospital for surgical intervention. Since the patient is symptomatic, with high-risk anatomy (malignant arterial course) and an increased risk of a major adverse cardiac event, the heart team recommended open-heart surgery to correct the RCA's anomalous origin, specifically reimplantation of the RCA to the right coronary sinus. Unroofing is generally performed in the young patient with a significant intramural length of the anomalous vessel. For separate orifices in the sinus of Valsalva, without an intramural course, reimplantation is most often suitable, and, as such, was performed in this patient. In February 2024, the surgical procedure was performed with cardiopulmonary bypass and myocardial cardioplegic arrest. The RCA was carefully resected as a button, while ensuring that the conal branch was preserved, after which it was anastomosed to the right coronary cusp.

**Figure 5:**
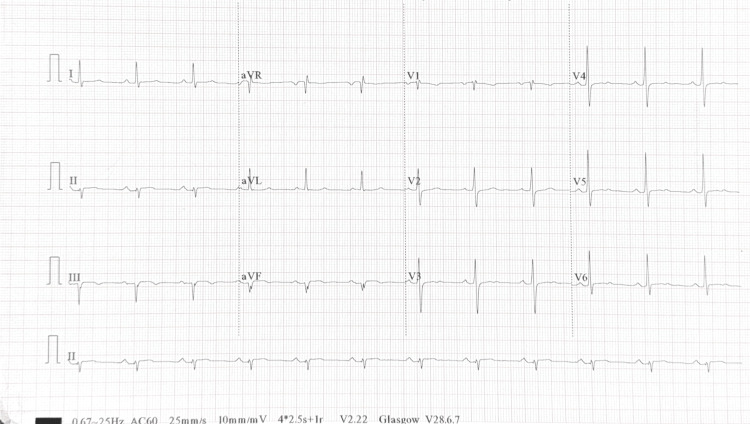
Post-operative ECG showing sinus rhythm with no signs of acute ischemia ECG, electrocardiogram

The patient's hemodynamic status remained stable. His ECG showed a normal sinus rhythm, with no evidence of ischemia during the postoperative course, and he was discharged on day 7 postoperatively (Figure 5).

**Figure 6:**
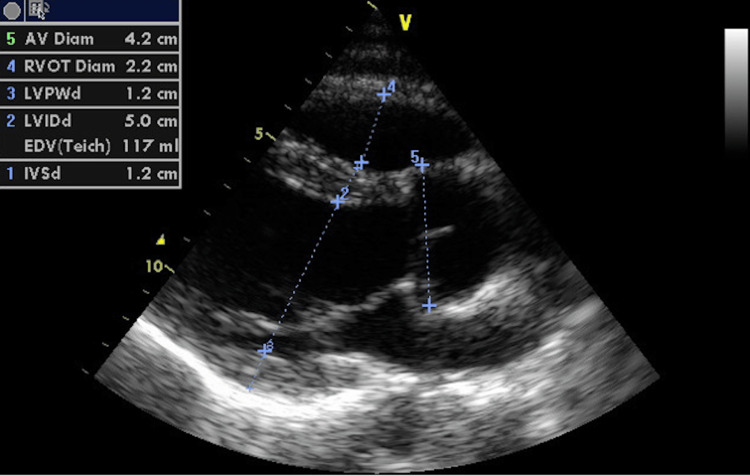
Echocardiogram: post-operative image of echocardiogram showing a non-dilated left ventricle with normal wall motion and systolic function, and no abnormalities

**Figure 7:**
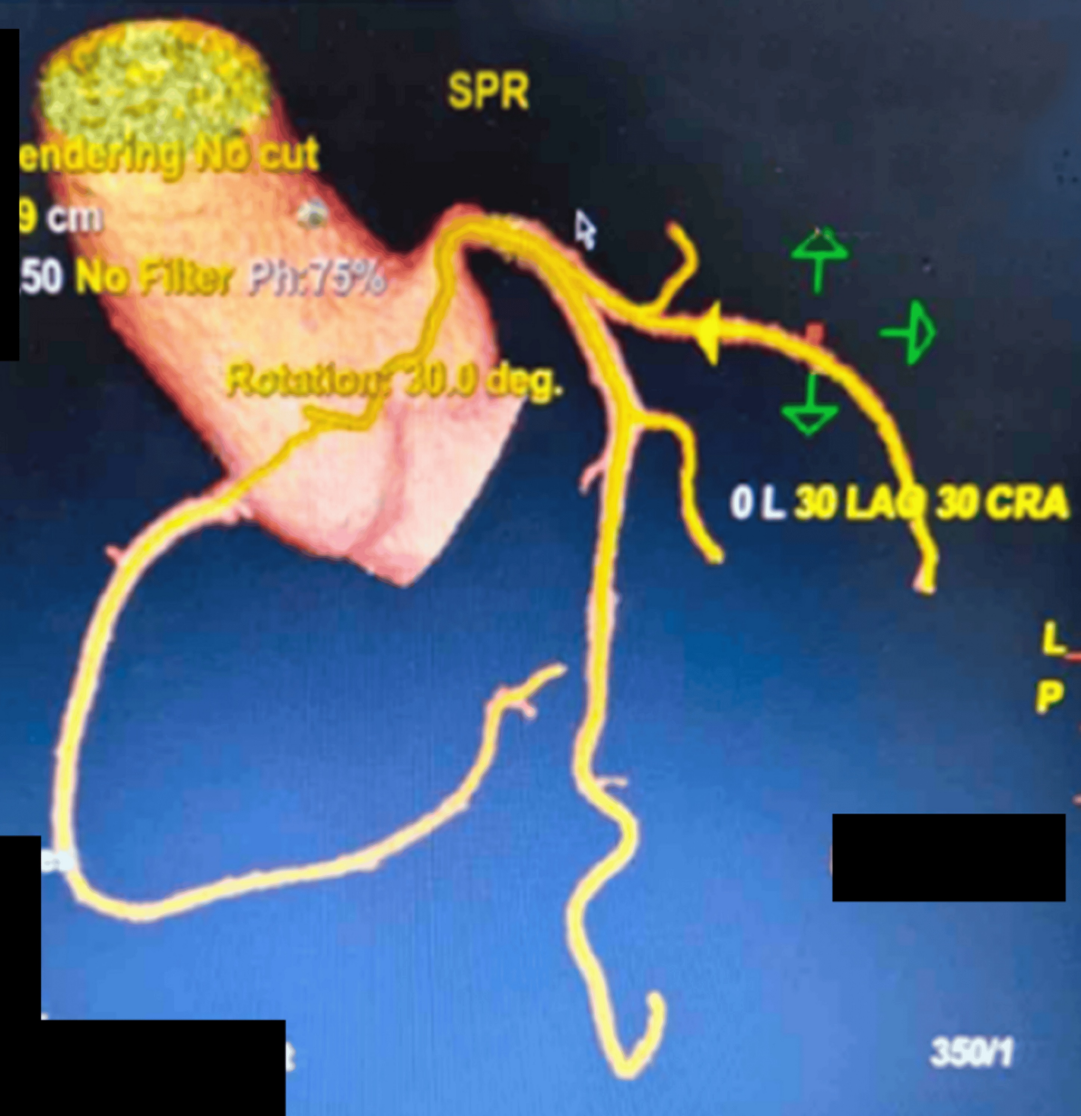
CT coronary angiogram: postoperative three-dimensional reconstruction of the reimplanted RCA in an isolated aortic view CT, computed tomography; RCA, right coronary artery

**Figure 8:**
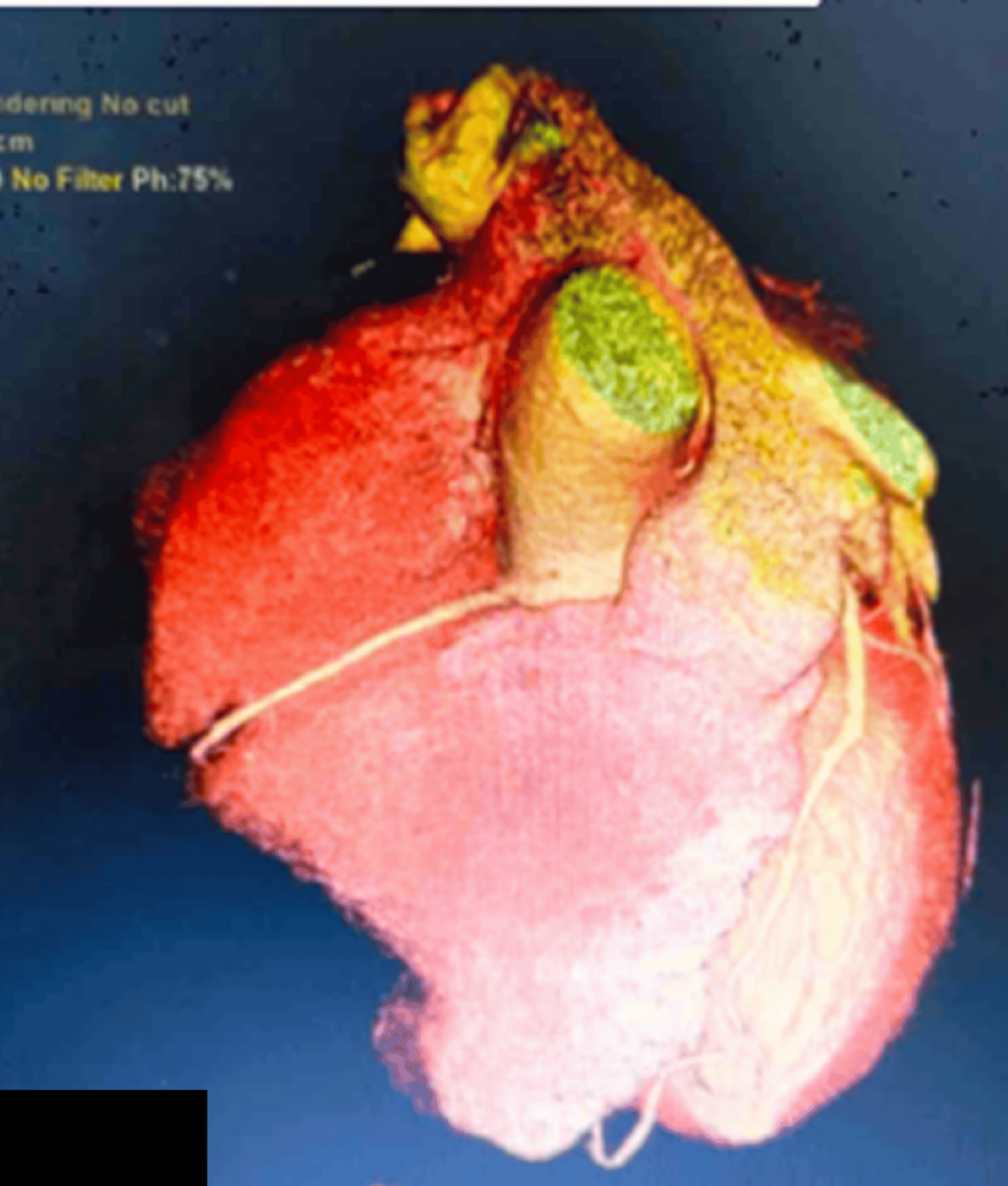
CT coronary angiogram: postoperative three-dimensional reconstruction of the reimplanted RCA CT, computed tomography; RCA, right coronary artery

The patient is regularly followed up in the outpatient cardiology clinic. In April 2024, an echocardiogram was performed, which revealed a non-dilated left ventricle, with normal wall motion and systolic function and no abnormalities (Figure 6). A computed tomography coronary angiogram (CTCA) revealed successful reimplantation of the RCA, with no significant stenosis or occlusion (Figures 7-8). The patient remained asymptomatic and is currently capable of engaging in regular activities.

## Discussion

AAORCA is a rare congenital anomaly with an estimated incidence of 0.1% to 0.9% [1,2]. The most common origin of the RCA is the left sinus of Valsalva, either from the left main coronary artery or a separate ostium [3,4]. Coronary anomalies represent the second leading cause of mortality in young athletes, after hypertrophic cardiomyopathy [3]. Approximately 15% to 34% of cases of sudden cardiac death in young individuals are attributed to these anomalies [4,5].

Patients can present with chest pain, palpitations, and syncope [2,6]. Complications include arrhythmias, myocardial ischemia, sudden cardiac arrest, and death, more commonly during or immediately after exercise [2]. High-risk features of an anomalous RCA are predisposing factors for ischemia [2,7]. These features include acute angulation of the artery, which leads to a slit-like orifice, causing kinking of the artery and subsequent coronary artery occlusion [2,7]. Other mechanisms include coronary spasm resulting from torsional movement and mechanical compression of the coronary artery between the pulmonary and aortic trunks during physical exertion [2,7]. Generally, patients do not report any symptoms, and this diagnosis is usually an incidental finding during echocardiography, cardiac catheterization, or provocative testing for other cardiovascular diseases [2]. 

Transthoracic echocardiography (TTE) is a non-invasive screening modality that is often used in patients with anomalous aortic origin of the coronary arteries (AAOCAs) to visualize the origin of vessels. However, a skilled operator is required for accurate diagnosis, and TTE has poor spatial resolution; therefore, it does not provide a detailed description of the anomaly and surrounding structures [6,8]. As such, CTCA and magnetic resonance coronary angiography (MRCA) are the preferred diagnostic tools because they provide high-resolution images compared to TTE. CTCA is preferred for anatomical assessment because it is readily available, has a rapid scan time, and offers high spatial resolution with calcium scoring, enabling the detection of coronary artery disease and revealing the dominant circulation of an anomalous origin of the RCA [9]. Early diagnosis and timely intervention can reduce the risk of sudden cardiac death, especially in symptomatic patients.

The choice of treatment for AAORCA is controversial. According to the 2020 ESC Guidelines for the management of adult congenital heart disease, surgical correction is recommended for AAOCAs in symptomatic patients who present with evidence of stress-induced myocardial ischemia or high-risk anatomy (Class I, Level of Evidence C) [6]. However, if a patient with AAORCA is asymptomatic and has no evidence of ischemia or high-risk anatomy, surgical correction is not recommended [6].

There are several surgical approaches for the repair of AAOCA, with the aim of eliminating the intramural course and any associated ostial narrowing of the anomalous artery by unroofing, ostioplasty, or reimplantation (Class I, Level of Evidence B) [2]. Unroofing is generally performed in young patients with a significant intramural length of the anomalous vessel [10]. For separate orifices in the sinus of Valsalva without an intramural course, reimplantation is most often suitable. Mobilization of the coronary button is necessary to allow direct reimplantation in the correct sinus and to avoid kinking and other distortions [10]. As such, in this case, there was successful reimplantation of the RCA into the right coronary sinus.

## Conclusions

In patients with symptoms of myocardial ischemia, coronary anomalies must be taken into consideration. AAORCA is a rare yet clinically significant congenital anomaly that may predispose patients to myocardial ischemia and sudden cardiac death, particularly during exertion. Although many cases remain asymptomatic and are discovered incidentally, recognition of high-risk anatomical features is essential to guide appropriate management. CTCA and MRCA are the recommended diagnostic modalities for evaluating patients with high-risk anatomical features and guiding surgical intervention. Early identification and surgical intervention in symptomatic individuals or those with high-risk anatomy can be lifesaving. The decision to pursue surgical correction should be individualized based on clinical presentation, anatomical findings, and evidence of ischemia. Ultimately, multidisciplinary evaluation and long-term follow-up remain crucial to optimize outcomes and reduce the risk of adverse cardiac events in patients with AAORCA. This case report highlights the successful reimplantation of the anomalous origin of the RCA.
